# Quantitative grey matter histological measures do not correlate with grey matter probability values from *in vivo* MRI in the temporal lobe

**DOI:** 10.1016/j.jneumeth.2009.05.001

**Published:** 2009-06-30

**Authors:** S.H. Eriksson, S.L. Free, M. Thom, M.R. Symms, L. Martinian, J.S. Duncan, S.M. Sisodiya

**Affiliations:** Department of Clinical and Experimental Epilepsy, UCL Institute of Neurology, London WC1N 3BG, UK

**Keywords:** Epilepsy, FSL, MRI, Neuronal density, SPM, Voxel-based morphometry

## Abstract

Voxel-based morphometry (VBM) is commonly used to study systematic differences in brain morphology from patients with various disorders, usually by comparisons with control subjects. It has often been suggested, however, that VBM is also sensitive to variations in composition in grey matter. The nature of the grey matter changes identified with VBM is still poorly understood. The aim of the current study was to determine whether grey matter histopathological measurements of neuronal tissue or gliosis influenced grey matter probability values that are used for VBM analyses.

Grey matter probability values (obtained using both SPM5 and FSL-FAST) were correlated with neuronal density, and field fraction of NeuN and GFAP immunopositivity in a grey matter region of interest in the middle temporal gyrus, in 19 patients undergoing temporal lobe resection for refractory epilepsy.

There were no significant correlations between any quantitative neuropathological measure and grey matter probability values in normal-appearing grey matter using either segmentation program.

The lack of correlation between grey matter probability values and the cortical neuropathological measures in normal-appearing grey matter suggests that intrinsic cortical changes of the type we have measured do not influence grey matter probability maps used for VBM analyses.

## 1 Introduction

Voxel-based morphometry (VBM) is a whole-brain semi-automated technique for characterising structural brain differences *in vivo* (Ashburner and Friston, 2000). VBM is now frequently used to study systematic differences in brain morphology in patients with various disorders, usually by comparisons with control subjects. VBM of segmented grey matter images has been useful in identifying subtle changes in brain structure in a variety of diseases associated with neurological and psychiatric dysfunction such as schizophrenia (Job et al., 2002), bipolar disorders (Lochhead et al., 2004), obsessive compulsive disorder (Valente et al., 2005), Tourette's syndrome (Ludolph et al., 2006; Garraux et al., 2006), Alzheimer's dementia (Ishii et al., 2005), migraine (Rocca et al., 2006), multiple system atrophy (Hauser et al., 2006), amyotrophic lateral sclerosis (Kassubek et al., 2005), and epilepsy (Woermann et al., 1999a,b; Keller et al., 2002, 2004; Bernasconi et al., 2004; Colliot et al., 2006; Bonilha et al., 2006; Mueller et al., 2006; Betting et al., 2006). The most commonly used softwares for VBM are SPM (currently SPM5) and FSL-FAST (Zhang et al., 2001). In the analyses, T1-weighted volumetric images are first segmented into grey matter, white matter and cerebrospinal fluid (CSF) tissue probability maps (Ashburner and Friston, 2005). Grey matter probability maps are non-linearly normalised to a common template, smoothed to produce grey matter concentration maps (GMC, also known as grey matter “density” maps) and modulated by the Jacobian of the non-linear transformation to produce grey matter volume maps (GMV). Univariate tests are then used at every voxel to study differences in GMC/GMV after correction for multiple comparisons (Mechelli et al., 2005b).

In epilepsy, VBM has been used to evaluate patients with idiopathic generalised epilepsy (Woermann et al., 1999b; Betting et al., 2006), temporal lobe epilepsy with and without hippocampal sclerosis (HS) (Woermann et al., 1999a; Keller et al., 2002, 2004; Bernasconi et al., 2004; Mueller et al., 2006) and focal cortical dysplasia (FCD) (Colliot et al., 2006; Bonilha et al., 2006). Most studies have found differences between patients and control subjects. Sometimes these changes are not confined to the presumed abnormal area such as the sclerotic hippocampus or clearly visible FCD, but also seen in areas distant from the obvious lesions.

It has been suggested that VBM is sensitive not only to differences in brain morphology between patients and control subjects, but also to variations in the composition of the grey matter. The nature of such changes is still poorly understood. The most common interpretation of reduction in GMC/GMV is atrophy of grey matter (Keller et al., 2002; Wessels et al., 2006) or neuronal loss (Mueller et al., 2006). An increase in GMC/GMV is often interpreted as thickened grey matter or a result of poor grey/white matter demarcation (Keller et al., 2002), that may be due to gliosis or malformations of cortical development. Improved understanding of the basis of these abnormalities is essential for the interpretation of future VBM studies. The aim of the current study was to evaluate if intrinsic histopathological characteristics of grey matter influence the segmentation of MRI that produces the grey matter images used for the VBM comparisons, by correlating neuronal and glial markers with grey matter probability values achieved within the grey matter from both SPM5 and FSL-FAST. The histopathological measurements were entirely within the grey matter ribbon. We did not make comparisons of these with VBM derived GMC/GMV, that may reflect the proportion of grey and white matter in a voxel.

## 2 Methods

### 2.1 Subjects

We studied 19 epilepsy surgery patients undergoing anterior temporal lobe resections at the National Hospital for Neurology and Neurosurgery who had pre-operative T1-weighted volume scans and in whom resected specimens were processed according to our protocol (Eriksson et al., 2005). The study was approved by the Joint Research Ethics Committee of the National Hospital for Neurology and Neurosurgery and Institute of Neurology, UCL. All patients gave written informed consent to participate in the study.

The age range of the subjects was 23–49 years (median 36 years) and 11 were male. Thirteen had a right anterior temporal lobe resection, and six had surgery on the left. The surgical procedure was performed after our standard presurgical work-up, including EEG-video telemetry, conventional MRI (T1- and T2-weighted, proton density and FLAIR), psychological and psychiatric assessments and multi-disciplinary case conference. Clinical and MRI details are summarised in Table 1.

### 2.2 MR acquisition

All scans were acquired on the same 1.5T GE Signa MR scanner (GE Medical Systems, Milwaukee, WI) using the standard circularly polarised quadrature birdcage coil. The parameters for the T1-weighted volume sequence were: TE/TR/TI, 4.2/15/450 (ms), number of averages 1; flip angle 20°; acquisition matrix 256 × 192; field of view 24 cm × 18 cm; 3/4 phase FOV, 124 contiguous 1.5 mm slices, giving a voxel size of 0.94 mm × 1.25 mm × 1.5 mm. This scan was used for both the MR:pathology correlation and the image segmentation.

### 2.3 MR:pathology visual correlation

**Fig. 1:**
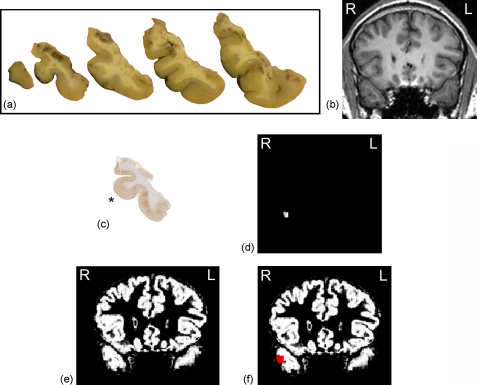
The resected temporal lobes (patient 12, right sided resection) were fixed and sliced in 5 mm tissue blocks perpendicular to the maximum linear extent of the superior temporal sulcus (a). The patients’ T1-weighted MRI scans were rotated and reformatted in an oblique coronal plane that matched the orientation of the pathology tissue blocks and the best-fit MRI slice for any one tissue block was identified (b, corresponding to tissue block approximately 1 cm from the tip of the temporal pole). The histopathological section 1–1.5 cm from the tip of the temporal pole was stained with NeuN (c). The cortical crown of the middle temporal gyrus identified (marked with *) and quantitative histopathological measurements were obtained from this area. The ROI on the MRI was determined using the previously established MR:pathology correlation to identify the MR slices that corresponded to the histopathological sections and the crown of the middle temporal gyrus was outlined using the same criteria as for the histopathological sections (d). The T1-weighted volume MR images were also segmented into grey (e) and white matter and CSF. The ROI was overlaid on the segmented grey matter class images/probability maps (f) to obtain the quantitative MR measurements.

All subjects had a standard anterior temporal lobe resection. During and after resection, specimens were processed according to our standard histopathological and co-registration protocols and digital images of the tissue blocks were co-registered with the pre-operative volumetric MRI data (Eriksson et al., 2005). Briefly, after fixation the resected temporal lobe was sliced in 5 mm tissue blocks perpendicular to the maximum linear extent of the superior temporal sulcus. The MR volume data were rotated and reformatted (using Analyze AVW 5.0, BIR, Mayo Clinic, MN) to 0.94 mm^3^ cubic voxels in an oblique coronal plane that matched the orientation of the pathology tissue blocks. These oblique coronal MR images were visually inspected in comparison to digital photographs of the tissue blocks. Using standardised criteria, the best-fit MRI slice for any one tissue block was identified, and matches for adjacent blocks of tissue ensued (Fig. 1). Our detailed co-registration methodology has been published (Eriksson et al., 2005).

### 2.4 Histopathology

Standard laboratory protocols were used to prepare resected tissue in sections stained for Nissl (cresyl violet/LFB), glial fibrillary acidic protein (GFAP; Dako, Cambridge, UK; polyclonal 1:1500) and neuronal nuclear antigen (NeuN; Chemicon, Temecula, CA, U.S.A., monoclonal 1:2000). An experienced epilepsy neuropathologist (MT) made a qualitative tissue assessment for clinical purposes. For quantitative histopathology the following sections were used: the standard 7 μm GFAP sections and additional 20 μm sections stained with NeuN. All sections were processed in the same batch to ensure uniform immunostaining, which is important for the field fraction analyses which rely on the intensity of the staining. A commercial image analysis system (Histometrix, Kinetic Imaging, Liverpool, UK), attached to a Zeiss Axioskop microscope, was used to obtain quantitative histopathology measures by a neurologist with extensive experience of quantitative neuropathology (SHE). A region of interest (ROI), comprising the cortical crown of the middle temporal gyrus 1–1.5 cm from the tip of the temporal pole, was analysed in each subject (Fig. 1). In all cases this region consisted of normal-appearing tissue as assessed qualitatively by the neuropathologist. In some patients there were neocortical abnormalities (see Table 1), but these were all distant from the ROIs. The quantitative histopathological measures were stereological counts of neurons and field fraction estimates of immunostaining for NeuN and GFAP, as previously described (Eriksson et al., 2006, 2007). The field fraction analyses were semi-automated. In brief, after manual setting of the RGB (red-green-blue) parameters representing immunopositive pixels, the computer software automatically estimated the number of immunopositive pixels as a proportion of the entire field. An average field fraction value was calculated for the whole ROI in each patient.

### 2.5 MRI quantitation

The ROI was also defined on the MRI T1-volume data used for the correlation with histopathology (reformatted to oblique coronal images with a voxel size of 0.94 mm^3^) using Analyze AVW 5.0 (BIR, Mayo Clinic, MN). The ROIs were outlined on each patient's T1-weighted scan and was not the same for each patient since the anatomy of the temporal lobes differed between the patients. The ROIs (cortical crown of the middle temporal gyrus) were extracted in subject space by manual drawing guided by visual inspection of the previously determined MR:pathology correlation. This step used the same criteria to characterise the limits of the gyral crown as were used in the quantitative neuropathology. The data from the initial MR:pathology correlations were used to determine which MR slices within the middle temporal gyrus corresponded to the ROI in each subject (Fig. 1). The manual outlining of the ROI was performed on the T1-weighted images rather than the segmented images to make use of the previous MR:pathology co-registration and ensure we were analysing the same areas on both MRI and histopathology in each patient.

The reformatted T1-weighted volume MRI data sets used for the MR:pathology correlations and definitions of the ROI were segmented in subject space using SPM5 (FIL, Wellcome Trust Centre for Neuroimaging, London, UK) and FSL-FAST (v. 3.53; Oxford Centre for Functional Magnetic Resonance Imaging of the Brain, Oxford, UK) (Fig. 1).

The standard recommended segmentation approach was used for each software. In SPM5, images were segmented using the defaults for the native space option. This produced tissue probability maps (grey matter, white matter and CSF images) that were in alignment with the T1-weighted images used for segmentation. In FSL-FAST, the brain was first extracted using BET (brain extracting tool) that eliminates all non-brain tissue automatically. Images were then segmented using the single-channel segmentation program. This produced grey matter, white matter and CSF images in subject space. Grey matter probability maps were converted from floating point to 8 bit format to enable comparisons with SPM output data.

No normalisation was needed for either procedure since the ROIs were outlined on the images in subject space that were then segmented and kept in subject space. In VBM analyses, smoothing is important to improve signal to noise ratio, to allow the images to conform more closely to a Gaussian field model and to increase the validity of statistical inference (Mechelli et al., 2005b). For the current study, however, no VBM comparisons were made, so images were kept unsmoothed to retain maximum image information.

Quantitative MR values were obtained from the SPM and FSL-FAST derived grey matter images/probability maps using a program written in-house that automatically obtains the value of each voxel within a defined region on the quantitative MR data set. The mean intensity value of all voxels within the ROI was calculated. We excluded any voxels which the segmentation algorithm classified as outside the grey matter, as shown by a voxel value of zero. Mean values were also obtained for the upper 90th-, 80th-, 70th-, 60th- and 50th-percentile of the quantitative values in each case, to evaluate the effects of exclusion of voxels that might partly be CSF or white matter.

For display purposes, the ROIs were overlaid in 3-dimensions on unsegmented reformatted T1-volume and segmented grey matter images using FSL View (http://www.fmrib.ox.ac.uk/fsl/fslview/index.html). The contour of the ROI was also outlined and then overlaid on axial MR images using DispImage 4.7 software (Plummer, 1992).

### 2.6 Statistical analyses

Spearman's rank correlation was used to assess any correlation between numbers of voxels outside the segmented grey matter between the two segmentation programs in each case. It was also used to investigate correlations between grey matter probability and quantitative neuropathological values. SPSS 11.0.0 was used for all statistical analyses. A significance level of *p* = 0.0014 was used in view of the number of comparisons being made (Bonferroni correction).

## 3 Results

After co-registration of histopathological tissue slices and MR images, ROIs could be outlined on stained histopathological sections and reformatted T1-volume images in all cases. The size of the ROIs varied due to anatomical differences in the size of the middle temporal gyrus.

Segmentation of the T1-weighted volume MRI was successful in all cases using both SPM5 and FSL-FAST.

**Fig. 2:**
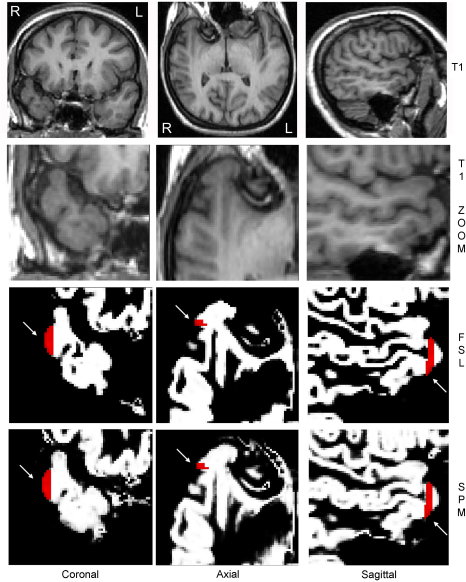
Top panels show full brain and zoomed T1-weighted volume images of right temporal lobe in patient 8, oblique coronal, axial and sagittal views. The third row shows grey matter probability maps (created using FSL). The fourth row shows grey matter class images (created using SPM5) of the right temporal lobe, same views as the zoomed T1-weighted images above. In the third and fourth rows the ROI (red) in the middle temporal gyrus is overlaid on the segmented images and is in the correct location (arrows). (For interpretation of the references to colour in this figure legend, the reader is referred to the web version of the article.)

**Fig. 3:**
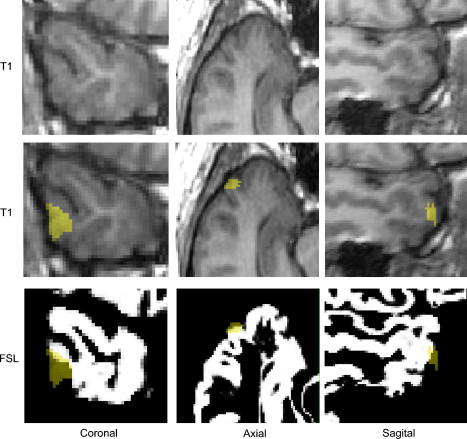
Top panel shows zoomed T1-weighted images and bottom panel shows grey matter class images (created using FSL-FAST) of the right temporal lobe in patient 15. The ROI (transparent yellow to allow visualisation of underlying tissue) is overlaid on the T1-weighted (in the correct location) and the segmented images (part of the ROI appears outside the brain, 57.6%). (For interpretation of the references to colour in this figure legend, the reader is referred to the web version of the article.)

Overlaying the ROIs, extracted from the T1-weighted MRI, on the reformatted T1-volume scans (used for ROI outlining and segmentation) showed the ROIs in appropriate locations (Fig. 2). Overlaying the ROIs on segmented grey matter images/probability maps did, however, show that at least part of the ROIs appeared outside the segmented tissue in a majority of the cases, 16/19 cases using SPM5 and all of the 19 cases using FSL-FAST (Fig. 3). This was verified by a proportion of the voxels within the ROIs exhibiting a value of zero (i.e. not containing any grey matter) in these cases. The proportion of voxels within the ROIs that appeared outside the grey matter was 0–13.1% for SPM5 and 3.0–57.6% for FSL-FAST. There was no significant correlation between the proportion of voxels outside the brain in each case between the two segmentation programs. No technical cause (such as mis-set image origins) was found to explain why part of the ROIs appeared outside the segmented grey matter images. Detailed inspection of image coordinates revealed that all images were in the same space and the ROIs were in the correct positions but that the most superficial parts of the unsegmented brains were not included in the segmented grey matter probability maps and part of the ROIs therefore appeared outside the grey matter.

As expected, in segmented grey matter probability maps, the voxels closest to the brain surface exhibited the lowest probability values using both segmentation programs. This was most pronounced using SPM5.

**Fig. 4:**
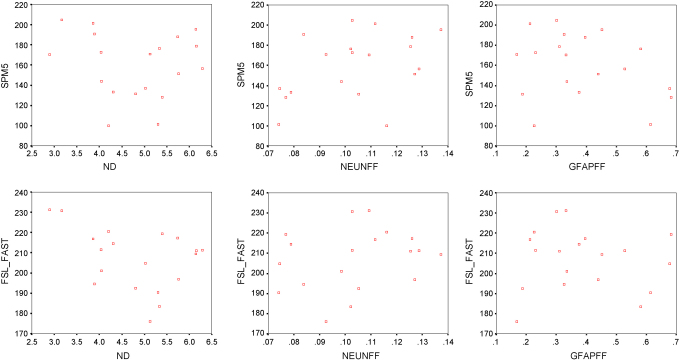
Scatter plots of SPM5 derived grey matter class values (top row) and FSL-FAST derived grey matter probability values (bottom row) (maximum 255) and neuronal density (ND, neurons × 10^−5^/μm^3^), NeuN field fraction (NEUNFF, %) and GFAP field fraction (GFAPFF, %).

There was no significant correlation between any quantitative histopathological measure (NeuN and GFAP field fraction and neuronal density) and mean grey matter probability values for either SPM5 or FSL-FAST at a corrected significance level of 0.0014 (Fig. 4 and Table 2). To explore if this was caused by the low values of voxels close to the brain surface, correlations were also performed using the mean values for the upper 90th-, 80th-, 70th-, 60th- and 50th-percentile probability values. There was no significant correlation between any of these probability values and quantitative neuropathological values.

## 4 Discussion

Whole-brain unbiased objective techniques (such as VBM) have been developed and used to characterise differences in brain morphology *in vivo* using structural magnetic resonance images. Some of the studies have suggested that VBM is sensitive to variations in composition, as opposed to the amount of grey matter, but the pathological bases of such changes are still poorly understood. In the current study, we correlated histopathological data with grey matter probability maps used for VBM analyses as a first step to improve our understanding of VBM results. The histopathological measurements were entirely within the grey matter ribbon. We did not make comparisons of these with VBM derived GMC/GMV, that may reflect the proportion of grey and white matter in a voxel.

Ashburner and Friston, in their original description of the SPM/VBM methodology implied that GMC (grey matter concentration) was not equivalent to neuronal density (Ashburner and Friston, 2000). Differences in GMC/GMV are obtained by comparing segmented grey matter probability maps between groups. In our study, we compared stereologically obtained neuronal densities with grey matter probability, and did not find any correlation between grey matter probability values and neuronal density, corroborating Ashburner and Friston's original proposition, even though our correlates were not with GMC or GMV. It is important that in the current study we were, however, only studying correlations within the cortical grey matter and cannot make any conclusions about tissue beyond this structure, or with comparisons taken at the level of a complete gyrus or lobe, in which neuronal density will be a complex composite measure, partly reflecting the relative proportions of grey and white matter.

There have been discussions on whether GMC or GMV might be related to differences in neuropil, neuronal size, dendritic or axonal arborisation (Mechelli et al., 2005a). The NeuN field fraction, a measure of the proportion of a tissue section that is occupied by immunopositive neuronal tissue, including at least parts of the somata, axons and dendrites, might be a good composite measure of such neuronal components. However, we did not find any correlation between SPM5 or FSL-FAST grey matter probability values and NeuN field fraction within the grey matter. This does not exclude that there might be a correlation between field fraction of other neuronal markers and grey matter probability. It has also been suggested that increased GMV could result from more folding as well as a thicker grey matter ribbon in the neocortex or poor grey/white matter demarcation (Mechelli et al., 2005b). Corroborating this will be complicated because of difficulties in confidently determining the border between grey and white matter on histopathological sections as well as MR images. It was not possible to address this in the current study, as our ROI was entirely within the cortical crown of the middle temporal gyrus. It has also been suggested that reduced GMC or GMV is caused by nerve cell loss and gliosis (Keller et al., 2002; Mueller et al., 2006; Wessels et al., 2006). We did not find any correlation between grey matter probability values and neuronal density or GFAP field fraction within the grey matter, for either segmentation approach.

It is of note in the current study that in a majority of the images the grey matter closest to the brain surface was not included in the segmented grey matter tissue probability maps for either SPM5 or FSL-FAST. This became obvious when part of the outlined ROI (comprising only the cortical crown in the middle temporal gyrus) that was in the correct location when overlaid on the T1-volume scans, appeared outside the brain on the segmented images (Figs. 3). This was initially thought to be a normalisation problem when we started the project using older versions of SPM that required normalisation before segmentation of images, but was still the case when images were kept in subject space using SPM5 and FSL-FAST. The reason for the most superficial parts of the grey matter not being classified as grey matter is not clear. Since no normalisation was performed, no *a priori* assumptions of tissue class were made and this might have contributed to segmentation problems. The proportion of grey matter that was not included in the segmented tissue varied between cases (0–13.1% for SPM5 and 3–57.6% for FSL-FAST). Smoothing of the images did not influence the placement of the ROIs. It is possible that the segmentation algorithm in some cases is too rigorous and hence fails to classify part of the cortex as grey matter. The segmentation may also be affected by susceptibility artefacts that are particularly common in the temporal lobes. The influence of these segmentation problems on VBM comparisons is not clear, but since the extent of superficial grey matter not included in the resulting grey matter probability maps varied between cases, it is possible that it might affect individual comparisons.

In summary, we did not find any significant correlation between grey matter probability values obtained using SPM5 or FSL-FAST and the chosen quantitative neuropathological measures in normal-appearing grey matter in individual patients. The lack of correlation between grey matter probability values and the cortical neuropathological measures obtained in this study suggests that the segmentation of MRI is not influenced by the intrinsic cortical characteristics we examined, but impact of other neuronal or glial markers cannot be excluded. Grey matter probability values may also be influenced by cortical signal intensity on T1-weighted images that may in turn be influenced by for example size of extra cellular space and water content as well as inflammation.

## Figures and Tables

**Table 1:** Clinical data, MRI and histopathological findings.

Patient	Age (years)/gender	Type of epilepsy	MRI findings	Histopathological findings
1.	36/M	R TLE	R temp tumour involving amygdala and hippocampal head	DNT
2.	38/F	L TLE	L temp calcified lesion, possible FCD	Ganglioglioma
3.	35/M	R TLE	Normal	Hamartia in white matter, approximately 2 mm diameter. Insufficient hippocampal material to allow confirmation of classic HS, but no evidence of neuronal loss or gliosis in CA1
4.	29/M	L TLE	L HS	HS, neuronal loss and gliosis in layer II-III
5.	23/M	R TLE	R temp tumour, possible DNT	DNT with adjacent cortical dysplasia, mild end folium sclerosis
6.	39/F	R TLE	R amygdala lesion, possibly tumour	HS
7.	34/M	L TLE	L HS	HS
8.	36/F	R TLE	R HS, R temp atrophy	HS, marked neocortical gliosis and neuronal loss (layer II)
9.	47/M	R TLE	R HS	HS, neuronal loss and gliosis in layer II
10.	26/M	L TLE	L HS	HS
11.	44/F	R TLE	Normal	Hippocampal gliosis—no classical HS
12.	36/M	R TLE	R HS	HS, neuronal loss and gliosis in layer II
13.	29/M	R TLE	R HS	HS
14.	37/M	L TLE	L HS	HS, neuronal loss and marked gliosis in layer II
15.	47/F	R TLE	R HS	HS
16.	32/F	R TLE	Slightly increased T2 in R hippocampus, no significant hippocampal atrophy	Hippocampal gliosis, insufficient hippocampal material to allow confirmation of classic HS
17.	33/F	R TLE	R HS	HS
18.	49/M	L TLE	L HS	HS
19.	41/F	R TLE	R HS	HS

F: female; M: male; L: left; R: right; TLE: temporal lobe epilepsy; HS: hippocampal sclerosis; DNT: dysembryoplastic neuroepithelial tumour.

**Table 2:** Quantitative MRI and histopathological data.

Patient	Mean grey matter probability value (SPM5), Max 255	Mean grey matter probability value (FSL-FAST), Max 255	Neuronal density (neurons × 10^−5^/μm^3^)	NeuN field fraction (%)	GFAP field fraction (%)
1.	133.1	214.6	4.31	7.9	37.65
2.	143.8	201	4.05	9.86	33.46
3.	100.0	220.5	4.2	11.62	22.57
4.	156.7	211.2	6.29	12.88	52.93
5.	101.1	190.4	5.31	7.42	61.40
6.	204.8	230.7	3.17	10.28	30.03
7.	131.4	192.4	4.81	10.52	18.77
8.	195.3	209.4	6.15	13.72	45.28
9.	187.9	217.3	5.74	12.60	39.54
10.	137.1	204.8	5.02	7.45	67.77
11.	178.9	211.1	6.16	12.54	30.97
12.	151.6	196.8	5.76	12.70	44.04
13.	170.4	231.3	2.89	10.92	33.28
14.	171.1	176.1	5.13	9.25	16.80
15.	190.9	194.6	3.89	8.39	32.60
16.	201.3	216.8	3.86	11.16	21.20
17.	176.5	183.4	5.34	10.22	58.15
18.	128.1	219.4	5.40	7.70	68.29
19.	172.8	211.4	4.04	10.27	23.03

NeuN: neuronal nuclear antigen; GFAP: glial fibrillary acidic protein.
